# Depression, suicidality, substance-use and associated factors among people living with HIV the COVID-19 pandemic in Uganda

**DOI:** 10.1371/journal.pone.0285310

**Published:** 2023-05-05

**Authors:** Godfrey Zari Rukundo, Edith K. Wakida, Christine K. Karungi, Jenipher Asasira, Edward Kumakech, Celestino Obua

**Affiliations:** 1 Department of Psychiatry, Mbarara University of Science and Technology, Mbarara, Uganda; 2 Department of Medical Education, California University of Science and Technology, Milpitas, CA, United States of America; 3 Office of Research Administration, Mbarara University of Science and Technology Mbarara, Mbarara, Uganda; 4 Department of Nursing and Midwifery, Faculty of Health Sciences, Lira University, Lira, Uganda; 5 Department of Pharmacology and Therapeutics, Mbarara University of Science and Technology, Mbarara, Mbarara, Uganda; The AIDS Support Organization, UGANDA

## Abstract

**Background:**

Mental disorders are common in people living with HIV (PLHIV) but they are often unrecognized and untreated. Furthermore, the COVID-19 pandemic has disrupted the already limited mental health services in low resource countries such as Uganda, and yet the extent to which the COVID-19 mitigation measures have affected the mental health of PLHIV is not fully known. We aimed to determine the burden of depression, suicidality, substance use and associated factors among adult PLHIV who were seeking care at two HIV clinics in northern and southwestern Uganda.

**Methods:**

We conducted a phenomenological qualitative and quantitative cross-sectional study among 431 PLHIV to determine the burden of depression, suicidality and substance-use disorders at two HIV clinics, at Lira Regional Referral Hospital and Mbarara Regional Referral Hospital in northern and southwestern Uganda respectively, during the COVID-19 lockdown. We used the Patient Health Questionnaire (PHQ-9) to assess for depression and suicidality, and the Michigan Assessment-Screening Test for Alcohol and drugs (MAST-AD) to assess for substance use disorder. We conducted descriptive statistics analysis to determine the burden of the disorders, and logistic regression to determine the associated factors. For the qualitative method we conducted in-depth interviews with 30 PLHIV and did thematic analysis.

**Results:**

Of the 431 PLHIV surveyed, mean age was 40.31 ± 12.20 years; 53.1% (n = 229) had depression; 22.0% (n = 95) had suicidality; and 15.1% (n = 65) had substance-use disorder. Female gender (PR = 1.073, 95%CI 1.004–1.148, P = 0.038), lack of formal education (PR = 1.197, 95% CI 1.057–1.357, P = 0.005), substance-use disorder (PR = 0.924, 95%CI 0.859–0.994, P = 0.034) and suicidality (PR = 0.757, 95%CI 0.722–0.794, p = 0.000) were associated with depression after adjusting for confounders. Further analysis showed that being female (PR = 0.843, 95% CI 0.787–0.903, P = 0.000*) and having depression (PR = 0.927, 95% CI 0.876–0.981, P = 0.009) and owning a large business (PR = 0.886, 95% CI 0.834–0.941, p = 0.000*) were significantly associated with having a substance-use disorder. Only depression was independently associated with suicidality after adjusting for confounding factors (PR 0.108, 95%CI 0.054–0.218, p = 0.000*). For the qualitative results, there were three apriori themes: a) Burden of depression, b) substance-use, and c) suicidality among the PLHIV during the COVID-19 containment measures.

**Conclusion:**

There was high prevalence of depression, suicidality and substance-use disorder in adult PLHIV in Uganda during the COVID-19 pandemic and the associated lockdown measures. The three mental health problems seem to have bidirectional relationships and gender has a lot of contribution to the relationships. Interventions aimed at any of the disorders should consider these bidirectional relationships.

## Introduction

Depression is one of the most common and yet unrecognized mental disorder among individuals with physical illnesses. Its presence is often associated with increased morbidity, long hospital stay, poor treatment adherence and poor quality of life [1]. When it is not assessed and treated appropriately it often leads to suicidality. In some cases, the affected individuals cope by using substances and other non-prescribed medications. Previous studies have reported high prevalence of mental health problems in chronic medical conditions and were associated with use of several medications, pain, stigma or shame. Some of these conditions include diabetes mellitus, hypertension, cancer, and HIV/AIDS [2]. Progress has been made in several countries to integrate mental health care into general health services. However, this is not yet realized in developing countries such as Uganda [3, 4]. Mental health problems in physical disease may also be complicated by emergency of new symptoms, delayed response to treatment or new comorbidities especially those that are either life threatening or terminal.

In 2019, while many countries were making progress with prevention and treatment of HIV/AIDS, COVID-19 emerged. COVID-19 is associated with several neurological and mental health problems [5, 6]. The mental health problems associated with the COVID-19 pandemic include anxiety disorder [7, 8], depression [9], suicidality [10, 11], substance use disorder [12–14] and domestic violence which can be a risk factor and a consequence of mental illness at the same time [15, 16]. In addition to the effect of SARCOV-2 itself, the infection preventive and control measures such as physical distancing, limited social gatherings and COVID-19 related stigma have brought additional challenges that individuals all over the world had to cope with. Many countries including Uganda instituted several lockdowns and other preventive measures as emergency measures without prior planning due to the emergency nature of COVID-19. Some of the preventive measures are uncomfortable and quite stressful and may trigger depression and negative coping mechanisms such as substances use or suicidality [17–21]. In Uganda, the first lockdown was instituted on March 18, 2020 with immediate effect for 32 days. The first case of COVID-19 in Uganda was confirmed on March 22, 2020. After this period, there was partial lifting of the lockdown in which private vehicles with up to three people were allowed to move. Public transport was also allowed to move at half capacity. Public gatherings (places of worship, markets and parties) remained limited. Keeping people in limited environments also escalated domestic violence [16]. Uganda registered its first COVID-19 death on July 24, 2020. By April 29, 2021, Uganda had registered 41,797 cases of COVID-19, and 342 deaths by April 30, 2021. These were aggregated COVID-19 cases and deaths for the entire Country including cases and deaths from northern and southwestern regions of Uganda. The number of new cases kept increasing probably due to laxity in implementing the containment and mitigation measures that had initially necessitated lockdown policies when WHO declared COVID-19 a global pandemic. The combination of the COVID-19 containment measures had not gone without downstream effects especially on access and utilization of healthcare services [22].

Studies conducted among people living with HIV/AIDS (PLHIV) in Uganda indicated high prevalence of mental health problems (such as depression, anxiety and suicidality) even before the COVID-19 pandemic [2, 23–31]. The COVID-19 pandemic has the potential of aggravating the existing burden of mental health problems among PLHIV because COVID-19 caused life disruptions such as closure of “non-essential” businesses that were sources of livelihood, travel restrictions and social isolation. As such, there are likely negative impact on life due to the increased levels of stress and anxiety [32–35]. In addition to closure of business and transport, some of the law enforcement officers in Uganda used excessive force and collided with the populace [6]. The COVID-19 pandemic has disrupted the already limited mental health services especially in developing countries, with patients avoiding seeking services in hospitals for fear of contracting the SARCOV-2 and also due to the restricted movements (public transport). According to the Ugandan Ministry of Health the consolidated guidelines for the management of HIV/AIDS includes the patient health questionnaire-2 (PHQ 2) to screen for depression. No other mental disorders are routinely assessed in HIV care. In the current study, depression was defined as a mental disorder characterized by the presence of sadness, loss of energy, loss of interest in pleasurable activities, feelings of guilt and low self-esteem, alterations in sleep patterns and the appetite, lack of concentration, and impairment in functioning [36]. We defined substance use disorder as a condition in which there is uncontrolled use of a substance despite its harmful consequences [37]. Likewise, suicidality in this study includes having suicidal ideation with or without suicidal plans or attempts [38]. The current study focused on suicidal ideation and suicidal attempts.

Just like care for other chronic medical conditions, HIV care requires regular and consistent attendance of clinic appointments and ARV refills [39]. The extent to which the COVID-19 mitigation measures have affected the prevalence of mental disorders among the PLHIV in Uganda is not fully known. However, previous studies have indicated the possibility of increase in mental health problems secondary to the COVID-19 pandemic [6, 40–42]. We aimed at determining the burden of depressive symptoms, suicidality, substance use and associated factors among adults living with HIV/AIDS seeking care at HIV clinics at Mbarara and Lira Regional Referral Hospitals in Uganda. The results of this study will inform mental health care planning as part of continuum of HIV/AIDS services in future pandemics or lockdowns.

## Methods

### Study site

This study was conducted at HIV clinics at Lira Regional Referral Hospital (LRRH) in northern Uganda and Mbarara Regional Referral Hospital (MRRH) in southwestern Uganda. The two regional referral hospitals were purposively selected so as to leverage the existence of the two HIV clinics which had well-established longitudinal cohorts of PLHIV and well-structured data systems. The two clinics offer comprehensive HIV services to children, adolescents and adults living with HIV. The services include ART enrolment and refills, adherence counselling, viral load measurements and community follow-ups. Both Mbarara and Lira regional referral hospitals are treatment centres for COVID-19 and the HIV clinic services were affected during the lockdown. Due to the ban on public transport, there was limited access to the HIV clinics for drug refills and recruitment into care. We aimed to make geographical comparisons because the capacities of the two populations to respond to the COVID-19 containment measures may have been different in the two regions. Northern Uganda went through decades of insurgency, psychological trauma, socio-economic, and educational disruptions which they have not fully recovered from, while southwestern Uganda has had relative peace with no disruption.

### Study design

**Fig 1:**
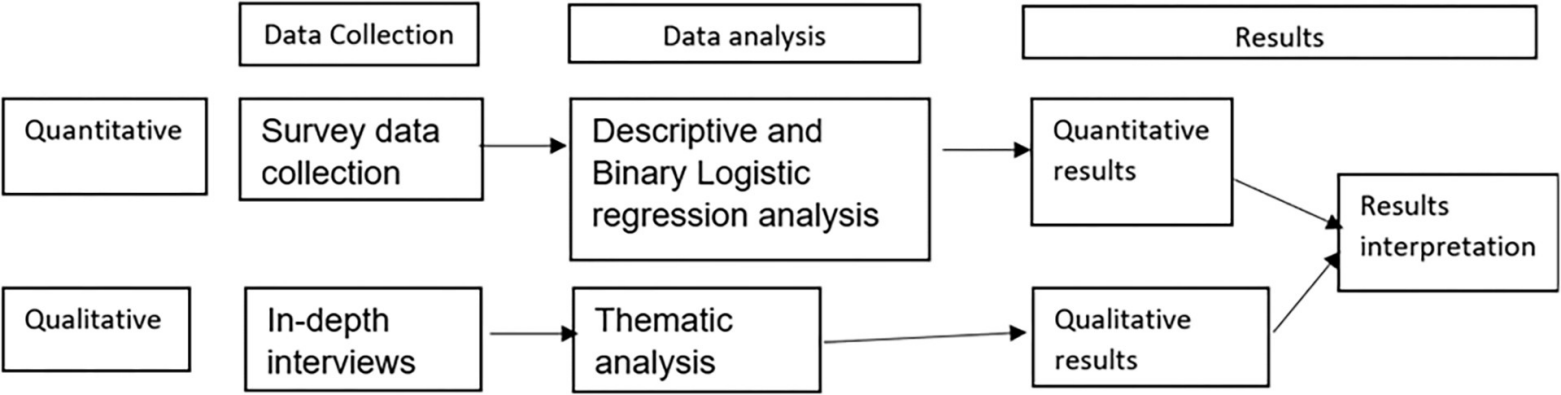
Qualitative and quantitative study design graphic representation.

We used a cross-sectional design with both qualitative and quantitative approaches to determine the burden of depression, suicidality and substance use disorders among PLHIV attending the two HIV clinics following the 2020 COVID-19 lockdown in Uganda. The data were collected in the March-April 2021. We started with a quantitative cross sectional survey in which we used the Patient Health Questionnaire (PHQ-9) [43, 44] to assess for depression and suicidality. The Michigan Assessment-Screening Test for Alcohol and drugs (MAST-AD) [45, 46] was used to assess for substance-use disorders. For the qualitative method we conducted in-depth interviews among PLHIV to explore the perceived burden of depression, suicidality, substance-use, lifestyle changes during the COVID-19 pandemic containment measures (Fig 1). We conducted the study during March 2021- six months after the partial lifting of the 2020 COVID-19 lockdown in Uganda when some limited public transport was allowed.

### Sample size and sampling

For the quantitative study, we determined the sample size by the sample size determination formula, N = Z^2^PQ/D2 [47], where: N = Sample size, Z = Standard deviation corresponding with 95% confidence interval in the standard normal distribution which is 1.96, P = 83.5% (0.835) was the estimated proportion of PLHIV in the target population with depression symptoms projected from before COVID-19 study in Uganda [38], D = 0.05 margin of error, Q = Estimated proportion of PLHIV in the target population without depression (1-p) = 16.5% (0.165). Thus, N = 1.96^2^ *0.835*0.165/0.05^2^ = 212 per study site. Given the 2 study sites, the design effect of 2.0 makes the total sample for the study of 424 PLHIIV. We adjusted the calculated sample by 1.8% to compensate for any missing data, giving the total sample of 431 participants divided equally between the two HIV sites. Given both HIV clinics see over ten thousand PLHIV and hence no significant differences in terms of PLHIV numbers, the equal distribution of the sample size between the two HIV sites was justifiable. At each of HIV clinics, we engaged data clerks to identify from the anti-retroviral therapy (ART) registers the PLHIV who were enrolled into care before January 2020. The study nurse then introduced the research team and the study objective and asked the potential participants to approach the research team for more information and possible participation. The individuals that went to the research team were given additional information about the study before asking them to consent. Using consecutive sampling (based on patient visits), we recruited the study participants at the MRRH and LRRH HIV clinics who consented to participate in the survey until we achieved the target sample size.

For the qualitative study, we conducted a total of 30 in-depth interviews (15 per facility) with PLHIV attending Lira and Mbarara Regional Referral Hospitals HIV clinics. The interviewees were purposively identified from the ART register while ensuring variations by age, gender, education level, employment status, distance from the ART clinic and duration on ART. The study nurse at the ART clinic introduced the study team to the selected interviewees when they visited the ART clinic on their clinic appointment days. The study objectives and procedures were introduced to the selected participants, informed consents were obtained and the consented participants were interviewed in a private and confidential space at the ART facility.

### Study variables

The independent variables were: age (years), gender, study site, tribe, religion, marital status, education and socio-economic status. The study had three outcome variables: depression, suicidality and substance use disorder. For depression, a score of 5 or more on the PHQ-9 met criteria for depression in varying levels of severity. Suicidality was assessed using the 9th question of the PHQ-9. The question required a yes or no. Anyone who answered yes was considered to have suicidality. Anyone who scored 4 or more on the MAST-AD, was considered to have a substance-use problem.

### Procedure for data collection

We engaged staff at the respective HIV clinics to help us identify the PLHIV who were enrolled into care before June 2020. Using consecutive sampling, we recruited the study participants at the two (MRRH and LRRH) HIV clinics until we achieve the target sample size. Depression was assessed using the Patient Health Questionnaire (PHQ-9) which is a 9-item tool with a possible minimum score of 0 and a maximum score of 27. The lower the total PHQ-9 score, the less likelihood of having depression, while the higher score indicated depression. If the total score was 0–4 the person was considered to have no depression. If the score was 5–9, there was mild depression; 10–14 moderate depression; 15–19 moderately severe depression and if the score was 20–27 there was severe depression. Suicidality was assessed using the 9th question of the PHQ-9. The PHQ-9 has previously been validated for use in Uganda by Nakku et al (2016) [48] and Miller et al (2021) [49] with the Cronbach alpha ranging between 0.68 and 0.94. Substance-use disorder was assessed using the Michigan Assessment-Screening Test for Alcohol and drugs (MAST-AD). The Michigan Alcoholism Screening Test for alcohol and drugs (MAST/AD) is a 25-item self-report that was developed by Selzer in 1971 [46] and later modified by Westermeyer et al 2004 [46]. This measures the severity and insight of drug and alcohol problems. On the MAST-AD, a score of 0–3 suggests no apparent substance-use problem; 4 indicates early substance-use; 5–7 aggravated substance-use and a score of 8 or more indicates chronic substance-use. In a previous study by Gibbs (1983), the Cronbach alpha for the MAST/AD ranged between 0.83 and 0.93 [50].

Both the quantitative and qualitative data were collected by trained research assistants in two local languages (Runyankore/Rukiga and Langi). The data collection tools were pre-tested to ensure that the participants would understand the questionnaires and that the tools would collect the right information. The participants in the pre-test were not included in the study.

For the qualitative approach, in-depth interviews were conducted using interview guides in two local languages (Langi and Runyankore/Rukiga in northern and southwestern Uganda respectively) by research assistants fluent in the two local languages. Each interview lasted between 30 to 45 minutes. The participants were asked to answer the following questions: 1) Tell me about any fears or concerns that you have had about COVID-19 when you are HIV positive? 2) How did the lockdown affect your ability to find purpose and meaning in life? 3) How did the lockdown affect your drinking of alcohol, smoking or use of drugs? 4) How did the lockdown affect your personal autonomy, and competence; believing your life and circumstances were under control? All the interviews were audio recorded and backed up with field notes. The audio recorded data were transcribed verbatim and later translated to English for analysis. The Runyankore/Rukiga and Langi speaking Research assistants read through the transcripts while listening to the audio recordings to check for correctness of information.

### Data analysis

Quantitative: We used SPSS Software v20 to enter and analyze the data. There were no missing data because the questionnaire was interviewer-administered. Results were presented as descriptive statistics for the burden of depression, suicidality and substance-use disorders in PLHIV. We performed Chi square test for associations of sociodemographic characteristics with depression. We also performed separate binary logistic regressions to determine factors associated with each of the outcome variables: depression, suicidality and substance use disorder with the level of statistical significance set at p ≤ 0.05. Using logistic regression analysis, we estimated the association between each of the outcome variables (depression, suicidality and substance use) and the various associated factors. The factors considered were: gender, level of education and socioeconomic status. The three outcome variables were considered as confounders for one another, and were included in the final models for each. In the current study, the Cronbach alpha for PHQ-9 was 0.89 while that of MAST-AD was 0.85.

Qualitative: The research assistants transcribed verbatim the audio-recordings in the local languages. The transcripts were then given to professional translators for translation into the English language while listening to the audio-recordings for clarity. We reviewed the English transcripts and sought clarification from the research assistants for the results which were not clear. Transcripts were independently coded (EKW and CKK) and disagreements resolved at each step. The coding process involved identification of similarities and differences through the participants’ narratives. Coding was done manually, grouping similar quotes together under broad themes derived from the respective study objectives while eliminating duplicates and ensuring inclusion of data from different sources. Data were analysed using thematic content analysis [51, 52]. CO and GRZ provided critique of the analysis and interrogated the coding to ensure defendable coding of data into relevant outcomes.

### Rigor of this study

In this study, credibility was ensured through establishing rapport with participants and allowing them to tell their story and experiences. Before data collection, we evaluated interview questions to ensure that they were open-ended. We achieved dependability through accurate documentation of the study processes. Adequate sampling and the purposive selection of the study participants were utilized so as to achieve transferability.

### Ethical considerations

The study was reviewed and approved by the Mbarara University Research Ethics Committee (MUST REC #03/01-21). Additional permission was sought from the Directors of the study hospitals to conduct the study at their HIV clinics. Before participating in the study, we obtained written informed consent from the participants. We obtained assent from participants who were not able to provide consent on their own (minors aged 15–17 years), but informed consent was obtained from their caregivers for the minors involvement in the study. The investigators and research assistants underwent training in responsible conduct of research and understood the importance of participant privacy, confidentiality and autonomy. At data entry participant identifiers (such as names, phone contacts) were included. Participants found to have severe mental health problems were referred to the metal health unit for further assessment and treatment.

## Results

### Participant socio-demographic characteristics

A total of 431 adults living with HIV participated in the study, giving a response rate of 100%. The sample comprised of 311 (72.2%) women and 120 (27.8%) men, median age of 40 years (range 16–91 years). The majority of participants were between 25–54 years (334, 77.5%), Anglican faith (209, 48.5%), married (215, 49.9%), and unskilled laborer (248, 57.5%) (Table 1).

**Table 1:** Sociodemographic characteristics of 431 PLHIV attending ART clinics of Lira and Mbarara regional referral hospitals in Uganda.

Variable	Description	Frequency N (431)	Percentage
**Age (years)**			
	15–24	44	10.2
	25–54	334	77.5
	55–64	39	9.0
	65+	14	3.2
**Gender**			
	Female	311	72.2
	Male	120	27.8
**Study site**			
	Lira	215	49.9
	Mbarara	216	50.1
**Tribe**			
	Lango	200	46.4
	Munyankore	162	37.6
	Muganda	25	5.8
	Other	44	10.2
**Religion**			
	Anglican	209	48.5
	Catholic	143	33.2
	Pentecostal	49	11.4
	Other	30	7.0
**Marital status**			
	Single	59	13.7
	Married	215	49.9
	Widowed	94	21.8
	Separated or Divorced	63	14.6
**Education**			
	No formal education	60	13.9
	Primary	157	36.4
	Secondary	150	34.8
	Tertiary	64	14.8
**Socioeconomic status**			
	Professional/civil servant	76	17.6
	Owner of large Business	10	2.3
	Crafts men and farmers	97	22.5
	Unskilled labour	248	57.5

*Socio-economic status was in five clusters

✓ Professionals with a university degree/senior civil servant

✓ Owners of large businesses with no university education

✓ Junior civil servants, primary school teachers, policemen, clerks.

✓ Craftsmen, farmers owning more than 3 acres of land, mechanics

✓ Small plot farmers, unskilled laborers

### Prevalence of depression, suicidality and alcohol/substance-use disorder among PLHIV attending HIV clinics at Lira and Mbarara regional referral hospitals

Of the 431 participants in the study, 53.1% (n = 229/431) had depression of which; 21.3% (92) had mild depression; Moderate depression 14.8% (n = 64); severe depression16.9% (n = 73). The prevalence of substance-use was 15.1% (n = 65/431) of these 1.6% (7/431) had early or middle substance-use, 5.8% (25/431) had aggravated substance-use and 7.7% (33/431) had chronic substance abuse or dependence. The prevalence of suicidal ideation was 22.0% (n = 95/431). Of those with suicidal ideation, 11.4% (49/431) had thought about hurting themselves for several days, 6.0% (26/431) had thought about hurting themselves more than half of the days and 4.6% (20/431) thought about hurting themselves almost every day (Table 2)

**Table 2:** Prevalence of depression, suicidality and alcohol/substance-use disorder among adult PLHIV attending HIV clinics at Lira and Mbarara regional referral hospitals.

Variable	Description	Total Participants N = 431					
		No depression n = 202 (46.9)	Depression n = 229 (53.1)	No Suicidality n = 336 (78.0)	Suicidality n = 95 (22.0)	No SUD n = 366 (84.9)	SUD n = 65 (15.1)
**Age (yrs)**	15–24	20 (4.6)	24 (5.6)	28 (6.5)	16 (3.7)	38 (8.8)	6 (1.4)
	25–54	159 (36.9)	175 (40.6)	265 (61.5)	69 (16.0)	278 (64.5)	56 (13.0)
	55–64	15 (3.5)	24(5.6)	33 (7.7)	6 (1.4)	37 (8.6)	2 (0.5)
	65+	8 (1.9)	6 (1.4)	10 (2.3)	4 (0.9)	13 (3.0)	1 (0.2)
**Gender**	Female	134 (31.1)	177 (41.1)	237 (55.0)	74 (17.2)	280 (65.0)	31 (7.2)
	Male	68 (15.8)	52 (12.1)	99 (23.0)	21 (4.9)	86 (20.0)	34 (7.9)
**Study site**	Lira	108 (25.1)	107 (24.8)	166 (38.5)	49 (11.4)	177 (41.1)	38 (8.8)
	Mbarara	94 (46.5)	122 (53.3)	170 (39.4)	46 (10.7)	189 (43.9)	27(6.3)
**Tribe**	Lango	98 (22.7)	102 (23.7)	154 (35.7)	46 (10.7)	164 (38.1)	36 (8.4)
	Munyankore	73 (16.9)	89 (20.6)	130 (30.2)	32 (7.4)	144 (33.4)	18 (4.2)
	Muganda	9 (2.1)	16 (3.7)	18 (4.2)	7 (1.6)	21 (4.9)	4 (0.9)
	Other	22 (5.1)	22 (5.1)	34 (7.9)	10 (2.3)	37 (8.6)	7 (1.6)
**Religion**	Anglican	94 (21.8)	115 (26.7)	161 (37.4)	48 (11.1)	178 (41.3)	31 (7.2)
	Catholic	73 (16.9)	70 (16.2)	110 (25.5)	33 (7.7)	118 (27.4)	25 (5.8)
	Pentecostal	24 (5.6)	25 (5.8)	44 (10.2)	5 (1.2)	44 (10.2)	5 (1.2)
	Other	11 (2.6)	19 (4.4)	21 (4.9)	9 (2.1)	26 (6.0)	4 (0.9)
**Marital status**	Single	24 (5.6)	35 (8.1)	37 (8.6)	22 (5.1)	52 (12.1)	7 (1.6)
	Married	111 (25.8)	104 (24.1)	175 (40.6)	40 (9.3)	174 (40.4)	41 (9.5)
	Widowed	38 (8.8)	56 (13.0)	78 (18.1	16 (3.7)	85 (19.7)	9 (2.1)
	Separated/Divorced	29 (6.7)	34 (7.9)	46 (10.7)	17 (3.9)	55 (12.8)	8 (1.9)
**Education**	No education	17 (3.9)	43 (10.0)	44 (10.2)	16 (3.7)	53 (12.3)	7 (1.6)
	Primary	63 (14.6)	94 (21.8)	114 (26.5)	43 (10.0)	130 (30.2)	27 (6.3)
	Secondary	80 (18.6)	70 (16.2)	121 (28.1)	29 (6.7)	126 (29.2)	24 (5.6)
	Tertiary	42 (9.7)	22 (5.1)	57 (13.2)	7 (1.6)	57 (13.2)	7 (1.6)
**Socio-economic status**	Civil servant	41 (9.5)	35 (8.1)	63 (14.6)	13 (3.0)	65 (15.1)	11 (2.6)
	Business Owner	8 (1.9)	2 (0.5)	7 (1.6)	3 (0.7)	10 (2.3)	0 (0)
	Craftsman/farmer	43 (10.0)	54 (12.5)	79 (18.3)	18 (4.2)	84 (19.5)	13 (3.0)
	Unskilled labourer	110 (25.5)	138 (32.0)	187 (43.4)	61 (14.2)	207 (48.0)	41 (9.5)

### Factors associated with depression among the PLHIV

At bivariate analysis; gender, level of education, socioeconomic status, substance-use disorder and suicidality were associated with depression. At multivariate analysis only female gender (PR = 1.073, 95%CI 1.004–1.148, P = 0.038), lack of formal education (PR = 1.197, 95% CI 1.057–1.357, P = 0.005), substance-use disorder (PR = 0.924, 95%CI 0.859–0.994, P = 0.034) and suicidality (PR = 0.757, 95%CI 0.722–0.794, p = 0.000) were associated with depression after adjusting for confounders. Tribe, religion, marital status and age had no significant association with depression among study participants (Table 3).

**Table 3:** Factors associated with depression among adult people living with HIV at Lira and Mbarara regional referral hospitals (N = 431).

Variable	Description	cPR	95% CI	P-Value	aPR	95% CI	P-Value
**Study site**	Lira^a^	1					
	Mbarara	0.957	0.900–1.018	0.162			
**Tribe**	Lango^a^	1.007	0.903–1.122	0.905			
	Munyankore	1.033	0.925–1.153	0.565			
	Muganda	1.093	0.940–1.272	0.247			
	Other						
**Religion**	Anglican^a^	0.949	0.847–1.064	0.370			
	Catholic	0.912	0.810–1.027	0.129			
	Pentecostal	0.925	0.803–1.064	0.274			
	Other	1					
**Marital status**	Single^a^	1.035	0.925–1.158	0.550			
	Married/ cohabiting	1.244	0.879–1.158	0.429			
	Widow/widower	0.799	0.937–1.147	0.489			
	Separated/Divorced	1					
**Gender**	Female	1.095	1.020–1.175	0.013	1.073	1.004–1.148	0.038
	Male^a^	1					
**Education**	No formal education^a^	1.278	1.145–1.425	0.000*	1.197	1.057–1.357	0.005
	Primary	1.190	1.0781.3314	0.001*	1.103	0.984–1.238	0.093
	Secondary	1091	0.9851.208	0.093	1.043	0.934–1.165	0.457
	Tertiary	1					
**Socio-economic status**	Professional/civil servant	0.938	0.861–1.620	0.149	1.032	0.941–1.131	0.503
	Owner of large Business	0.771	0.625–1.023	0.015	.807	0.642–1.014	0.066
	Crafts men and farmers	1.000	0.928–1.78	0.997	1.049	0.980–1.123	0.167
	Unskilled labour^a^	1			1	.	.
**Age (years)**	15–24	1.082	0.881–1.328	0.452			
	25–54	1.067	0.887–1.283	0.493			
	55–64	1.131	0.922–1.387	0.239			
	65+	1					
**Substance-use disorder**	No	0.918	0.849–0.993	0.032	0.924	0.859–0.994	0.034
	Yes	1			1	.	.
**Suicidality**	No	0.743	0.708–0.778	0.000*	0.757	0.722–0.794	0.000
	Yes	1			1		

^a^ Reference value

* Reflects statistically significant results at p ≤0.05

### Factors associated with substance-use disorder

Further analysis showed that being female (PR = 0.843, 95% CI 0.787–0.903, P = 0.000*) and having depression (PR = 0.927, 95% CI 0.876–0.981, P = 0.009) and owning a large business (PR = 0.886, 95% CI 0.834–0.941, p = 0.000*) were significantly associated with having a substance-use disorder (Table 4).

**Table 4:** Factors associated with substance use disorder among adult people living with HIV at Lira and Mbarara regional referral hospitals (N = 431).

Variable	Description	cPR	95% CI	P-Value	aPR	95% CI	P-Value
**Study site**	Lira^a^	1.045	0.987–1.109	0.131			
	Mbarara	1					
**Tribe**	Lango^a^	1.018	0.918–1.129	0.735			
	Munyankore	0.959	0.865–1.063	0.421			
	Muganda	1.001	0.857–1.169	0.992			
	Other	1					
**Religion**	Anglican^a^	1.013	0.903–1.137	0.823			
	Catholic	1.037	1.168	0.556			
	Pentecostal	0.972	1.110	0.678			
	Other	1					
**Marital status**	Single^a^	0.993	0.895–1.101	0.888			
	Married/ cohabiting	1.057	0.970–1.151	0.206			
	Widow/widower	0.972	0.888–1.065	0.545			
	Separated/Divorced	1					
**Gender**	Female^a^	0.857	0.799–0.919	0.000	0.843	0.787–0.903	0.000*
	Male	1					
**Education**	No formal education^a^	1.007	0.911–1.113	0.898			
	Primary	1.056	0.970–1.151	0.208			
	Secondary	1.048	0.960–1.139	0.306			
	Tertiary	1					
**Socio-economic status**	Professional/civil servant^a^	0.982	0.907–1.064	0.661	0.964	0.893–1.039	0.336
	Owner of large Business	0.858	0.825–0.893	0.000	0.862	0.814–0.912	0.000*
	Crafts men and farmers	0.973	0.906–1.045	0.457	0.950	0.885–1.020	0.156
	Unskilled labour	1					
**Age (years)**	15–24^a^	1.061	0.909–1.238	0.455			
	25–54	1.090	0.956–1.242	0.196			
	55–64	0.981	0.851–1.131	0.793			
	65+	1					
**Suicidality**	No	1					
	Yes	0.947	0.879–1.021	0.155			
**Depression**	No	0.941	0.888–0.997	0.040	0.927	0.876–0.981	0.009
	Yes	1					

^a^ Reference value

* Reflects statistically significant results at p ≤0.05

### Factors associated with suicidality

For suicidality, only depression remained statistically significant after adjusting for confounding factors (PR 0.108, 95%CI 0.054–0.218, p = 0.000*) (Table 5).

**Table 5:** Factors associated with suicidality among adult people living with HIV at Lira and Mbarara regional referral hospitals (N = 431).

Variable	Description	cPR	95% CI	P-Value	aPR	95% CI	P-Value
**Study site**	Lira^a^	1.070	0.750–1.527	0.708			
	Mbarara	1					
**Tribe**	Lango^a^	1.012	0.555–1.846	0.969			
	Munyankore	0.869	0.464–1.627	0.666			
	Muganda	1.232	0.536–2.831	0.623			
	Other	1					
**Religion**	Anglican^a^	0.766	0.420–1.395	0.383	0.874	0.519–1.472	0.611
	Catholic	0.769	0.412–1.434	0.409	0.994	0.582–1.698	0.982
	Pentecostal	0.340	0.126–0.919	0.034	0.431	1.075	0.071
	Other	1					
**Marital status**	Single^a^	1.382	0.818–2.333	0.226			
	Married/ cohabiting	0.689	0.421–1.129	0.139			
	Widow/widower	0.345	0.345–1.153	0.135			
	Separated/Divorced	1					
**Gender**	Female^a^	1.360	0.879–2.104	0.168			
	Male	1					
**Education**	No formal education^a^	2.438	1.079–5.510	0.032	1.350	0.647–2.817	0.424
	Primary	2.504	1.190–5.270	0.016	1.563	0.800–3.051	0.191
	Secondary	1.768	0.817–3.824	0.148	1.436	0.720–2.862	0.304
	Tertiary	1					
**Socio-economic status**	Professional/civil servant^a^	0.695	0.405–1.194	0.188			
	Owner of large Business	1.220		0.689			
	Crafts men and farmers	0.754	0.471–1.208	0.240			
	Unskilled labour	1					
**Age (years)**	15–24^a^	1.273	0.509–3.180	0.606			
	25–54	0.723	0.308–1.699	0.457			
	55–64	0.538	1.631	0.274			
	65+	1					
**Substance-use disorder**	No	0.710	0.463–1.090	0.117			
	Yes	1					
**Depression**	No	0.104	0.052–0.210	0.000*	0.108	0.054–0.218	000*
	Yes	1					

^a^ Reference value

* Reflects statistically significant results at p ≤0.05

### Qualitative results

We interviewed 30 adult PLHIV aged between 26–59 years, comprising of 11 (36.6%) males and 19 (63.4%) females (Table 6). We present the result under the three *apriori* themes: a) Burden of depression, b) substance-use, and c) suicidality among the PLHIV during the COVID-19 containment measures.

**Table 6:** Participant demographic characteristics for the qualitative study.

**Age categories (years)**	**Age**	**Description**	**FREQUENCY (%)**
		26–35	10 (33.3)
		36–45	8 (26.7)
		46–55	8 (26.7)
		56 and above	4 (13.3)
		**Total**	**30 (100)**
**Gender**	**MRRH**	M	7 (23.3)
		F	8 (26.7)
	**LRRH**	M	4 (13.3)
		F	11 (36.7)
	**Total**		**30(100)**

#### Theme 1: Burden of depression among PLHIV

On this theme, we particularly wanted to find out what fears or concerns the participants may have had as PLHIV during the lockdown period in the country. Respondents generally thought that the pandemic would spell the end of the world for them especially where information was circulating that PLHIV would likely suffer worse from the pandemic.

*Question (Qn). Tell me about any fears or concerns that you have had about COVID-19 when you are HIV positive?*

*‘Yah for me I thought that it would be the end of the world for me, because, first of all, I wasn’t on any treatment and HIV also being a virus that is very deadly when you are not on treatment, and there is also the COVID-19 virus, I became very confused and worried …… so I felt I was in danger’. (**38-year female-1, LRRH)***

*‘…. we people living with HIV virus have a lot of fear even now, we are still having fear that CORONA may still kill us, that is the fear in my heart because they say that when it gets you when you are living with HIV virus, you do not take long, and so I was really distressed’. (**33-year male, MRRH)***

During the lockdown, some participants found no purpose or hope in life and so resorted to doing things they would normally not do in life, such as, engaging in commercial sex work and/or taking alcohol.

*Qn. Tell me how the lockdown may have affected your ability to find purpose and meaning in life?*

*‘Corona has brought changes, in that, I now know that any time even if am still alive, I know that I have no one to help me, because there is no where I can get help, I lost meaning in life. Another thing is that it made me to know that life does change, that is, I started selling myself (sex-work), I started doing many things, somethings that I thought I would not do in my life, but when I was doing that, my mind tells me that I am doing wrong things, …I never thought I would start taking alcohol but when the lock down came, I started taking alcohol, …when I take it (alcohol), it makes me to forget about all my stress…’ **(35-year female, LRRH)***

Other participants were psychologically distressed as they felt abandoned due to lack of family support during the lockdown, and in some cases, this resulted in marital separation or being chased away from their martial homes by in-laws.

*Qn. Tell me how the lockdown may have affected your optimism, providing and receiving support from family, friends and others?*

*‘…there was support only during the time when my husband was alive. During the lockdown my husband passed on when he could not access his drugs (ARTs), because when the lockdown started, he was working from afar, I collected for him his drugs but how to send them to him was very difficult. He then became very ill… and he lost his life. But when my husband passedon, people from his home (the in-laws) changed their mind against me, they chased me out of the home he left me in,.. and now I am renting a house’. **(39-year female, LRRH)***

#### Theme 2: Substance-use disorder among PLHIV during the COVID-19 containment measures

In this theme, we explored substance-use disorder among the PLHIV, where we found that some participants who were not initially consuming substances such as alcohol, cigarettes and illicit drugs resorted to using them following the COVID-19 containment measures while the ones who were already on them intensified use in a bid reduce the stress of the lockdown.

*Qn. Tell me how the lock down may have affected your drinking of alcohol, smoking or use of drugs?*

*‘Before lockdown I wasn’t taking anything (alcohol), but it was during the lockdown when I lost hope and I couldn’t sleep… I would be so stressed… I started bit by bit drinking alcohol to get sleep and relax in the night’. (**37-year female participant LRRH)***

*‘During COVID19 there was no job so my husband joined bad groups where they drink alcohol, play cards and smoke drugs (khat & Marijuana). When he came back home, he could fight us, at times even chases me out of the house, I would go and sleep at the neighbors’ homes’. (**30-year female, LRRH)***

*‘I had things to do (work) before lockdown, but during lockdown, there was no work, sometimes I would spend my day at home just chewing it (khat), … you understand. So, during lockdown, the way I was using khat increased compared to before lockdown’. **(29-year male, MRRH)***

The lockdown paradoxically brought about some positive lifestyle changes, where some participants with no source of income and no money to spend stopped substance-use and even avoided socializing with friends for fear of contracting the COVID-19 virus.

*‘Before lock down I used to take alcohol but when lock down came, I stopped taking alcohol, because the way I would get money to buy alcohol was not there, not only that, I was even fearing to meet with my friends because I thought I may contract this disease (COVID-19), so it made me to stop taking alcohol, up to now, I thank God for what he did, I thank him a lot’. **(32-year male, LRRH)***

#### Theme 3: Suicidality

In this theme we found that the lockdown caused confusion, lack of personal control, despair and suicidal feelings among the participants. The participants were asked to respond to a statement: ‘‘Tell me how the lockdown may have affected your personal autonomy, and competence; believing your life and circumstances were under control”. Here below are some of the quotes from the participants:

*‘With the lockdown you see, my mental health…like I told you, I became very confused and helpless in mind, to a point where I would even think of committing suicide…because that time there was no social gathering and yet I am this person who has been living in the church’. **(38-year female MRRH)***

*‘Aaah! (sighs), for me during the lock down, I started thinking negatively, …so living with HIV, always I thought that anytime I should just die, so that I rest with these earthly problems, …. Negative thought was too much, every time I would only think of dying’. **(33-year female, LRRH)***

In addition to suicidality, the participants reported feelings of hopelessness which is a common risk factor for suicide. Here below are some of their responses:

*‘During lock down, I had already accepted to take life just like way change is, either to live or to die. I had already lost hope this disease has come to finish us …. So, there was no hope in life; we were only staying like aaa, if we enter another day it is ok; if we do not enter, it is also ok, because we do not have any hope’. **(56-year male, LRRH)***

*‘.. Mentally I became, you know, I can say I became retarded because I would not think positively of how I could achieve other things in life, and what I could do with the little energy I had. In fact, I would not think of the future. I lost hope’. (**38-year female, LRRH)***

## Discussion

In this study, we determined the burden of depression, suicidality and substance-use among 431 adults living with HIV/AIDS and seeking care at HIV clinics in Mbarara and Lira Regional Referral Hospitals in Uganda, where the prevalence of depression was 53.1%, substance-use disorder was 15.1% and suicidality was 22.0%.

### Prevalence of depression and associated factors

Compared to other recent studies on depression among individuals living with HIV, the prevalence of depression at 53.1% in our study was quite high, and although we used a screening tool to assess depression. The 14.8% with moderate depression and the 16.9% with severe depression required medical intervention. This prevalence is higher than what has been reported by pre-COVID-19 studies in similar study populations. For instance, Kinyanda et al reported a prevalence of 8.1% in one study [27] and in another study [24] they reported a prevalence of 14%, and several other studies by Hatcher et al [53], Kiene et al [54], Kaida et al [55] and Velloza et al [56], all reported lower prevalence of depression. In our study we would have expected prevalence in the same range, however the difference could be attributed to the challenges associated with the COVID-19 pandemic, where the lockdown situation may have contributed to more participants feeling depressed as was found during the in-depth interviews. The high prevalence of depression in our study is similar to reports from older studies in Uganda among HIV positive populations. For instance, pre-COVID-19 study by Kaharuza et al [57] reported 47% prevalence of depression, and Nakasujja et al [58] also reported 54% prevalence of depression. A recent study by Wagner et al 2022 reported an increase in depression in the post-COVID period [59]. A more recent study by Beyamo et al (2020) in Ethiopia [60] also reported a prevalence of 50.5% slightly lower than that we have found in this study although the studies were done in pre-COVID-19 era. Our study findings are also similar what has been reported by Tee et al (2020) in the Phillipines in which 16.9% had moderate to severe depressive symptoms [61].

In our study, the odds for depression were significantly increased by substance-use disorder, suicidality, gender and having low or no education. Pre-COVID-19 studies also found an association between gender [62–64], low levels of education [65] and depression which showed higher odds of depression among people living with HIV.

### Prevalence of suicidality and associated factors

In this study we found that the prevalence of suicidal ideation of 22.0%. We further found that more than half (10.6%) of the participants had thoughts of suicide for several days. These compares with rates of suicidality and suicide ideation found among people living with HIV in many other pre-COVID-19 studies with the prevalence ranging from a high of 52% in Australia [66], 31% in the United Kingdom [67], and 23% in France [68]. These confirm the finding from a systematic review which showed that living with HIV/AIDS is generally associated with suicidality or dying from suicide [69]. This was further elaborated by participants during the in-depth interviews who reported that living with HIV combined with the COVID-19 lockdown situation led them to want to commit suicide [70]. The finding in our study is however higher than prevalence reported from pre-COVID-19 studies in Uganda among a similar study population. For instance, Rukundo et al reported a prevalence of 10% in a study conducted at Mbarara Regional Referral Hospital and TASO HIV clinics in Mbarara city [2, 23]. Similarly, Kinyanda et al [26] reported a prevalence of suicidality of 7.8% and 2.8% in another study [24], while Mugisha et al reported a prevalence of 12.1% in post-conflict region of northern Uganda [71] all below the prevalence in our study. While the population of study were similar in these Uganda studies, we want to believe that the high prevalence in our study could have been due to the COVID-19 pandemic and the associated containment measures.

Although we had fewer male participants, we still found that being male had higher odds of suicidality, which was in contrast to a study in Ethiopia where the female PLHIV had higher odds of suicidality [72]. Our study did not show any association between religion and suicidality, although other studies had reported association between religion and suicidality [73, 74].

### Prevalence of substance-use disorders and associated factors

It is common to find alcohol use among individuals with HIV especially in developing countries especially in sub-Saharan Africa [75–77]. The prevalence of substance-use in our study was 15.1% which was similar to what had been from pre-COVID-19 from Uganda reported by Thakarar et al [29], although our study found a lower prevalence than that reported from pre-COVID-19 study by Chander et al [78]. We found that gender was associated with substance-use disorder, where being female was protective against substance-use disorder which is similar to what has been reported from pre-COVID-19 Nigerian study where female gender was protective against alcohol use disorder [76], or in the USA, where the male gender was associated with increased risk of substance-use disorder [79]. Our finding however shows a much lower prevalence of substance use disorder than the 30.1% hazardous alcohol use that was reported by Tran et al in Vietnam [80]. The lower prevalence of substance use disorder from our study could be due to the lockdown of bars and night clubs where alcohol is mostly consumed.

Depression often has a bidirectional relationship with substance-use [81, 82], as we did find that having depression had higher odds for substance-use and vice versa. This relationship has also been reported by other pre-COVID-19 studies [76]. Whereas people with depression may cope by using/misuse of substances, continued use of substance also puts the user at the risk of developing depression. From the in-depth interview participants did reveal that they took to use of alcohol or drugs as a means of coping with the distress of the lockdown or because they felt depressed. In addition, depression and substance-use disorder may also co-exist in the same individual without a causal relationship [83, 84]. This being a cross-sectional study, we were not able to determine the direction of the relationship between substance use and depression, where a future prospective study may be necessary.

Furthermore, the participants reported that they were psychologically distressed and felt abandoned. The COVID-19 related lockdown caused confusion, lack of personal control, despair and suicidal feelings among the participants. This could have been avoided by preparing the population for the lockdown before instituting it. This would have helped the individuals in coping with the associated challenges. Although the COVID-19 lockdown was largely negative, it brought about some positive lifestyle changes, where some participants with no source of income and no money to spend stopped substance-use and even avoided socializing with friends for fear of contracting the COVID-19 virus. This lifestyle change was also necessary for prevention of further spread of COVID-19.

#### Implications for HIV care

There is need for regular screening for depression, suicidality and substance-use among individuals living with HIV and AIDS. During this period of the COVID-19 pandemic, individuals need to be asked about how they are coping with the COVID-19 pandemic and its associated containment measures.

#### Limitations

This is a cross-sectional study and we are not able to compare the periods before, during and after the COVID-19 lockdowns. The fact that this study was conducted during the COVID-19 pandemic, it would have been good to compare the burden of mental problems before and during the pandemic.

### Conclusions

There was high prevalence of depression, suicidality and substance-use disorder among individuals living with HIV in Uganda during the COVID-19 pandemic. The three mental health problems seem to have bidirectional relationships gender has a lot of contribution to the relationships. Interventions aimed at any of the disorders should consider these bidirectional relationships. In addition, the ministry of health should consider integration of mental health into responses for future pandemics and other emergency situations so as to reduce the negative effects on mental health.
